# Health-Related Quality of Life and Social Outcomes in Adolescents and Young Adult Survivors of Childhood Cancer: A Single-Center Case–Control Study from Crete, Greece

**DOI:** 10.3390/reports8040207

**Published:** 2025-10-17

**Authors:** Ioannis Kyriakidis, Iordanis Pelagiadis, Nikolaos Katzilakis, Margarita Pesmatzoglou, Maria Stratigaki, Stylianos Megremis, Eftichia Stiakaki

**Affiliations:** 1 Department of Pediatric Hematology-Oncology & Autologous Hematopoietic Stem Cell Transplantation Unit, University Hospital of Heraklion & Laboratory of Blood Diseases and Childhood Cancer Biology, School of Medicine, University of Crete, 71003 Heraklion, Greece; kyriakidis@med.uoc.gr (I.K.); ipelagiadis@icloud.com (I.P.); katzilaher@yahoo.gr (N.K.); pesmatzoglou_m@hotmail.com (M.P.); mstratigaki@hotmail.com (M.S.); 2 Radiology Department, University Hospital of Heraklion, 71110 Heraklion, Greece; stylmegremis@yahoo.com

**Keywords:** childhood cancer survivors, adolescents and young adults, quality of life, late effects, social outcomes

## Abstract

Background: Recent advances in childhood cancer treatment and increased survival rates have led to a growing number of adolescents and young adults (AYAs) who are survivors of childhood cancer (CCSs). This study aimed to examine health status, health-related quality of life (HRQoL), and social outcomes in AYA CCSs. Methods: Sixty-two AYAs who were CCSs (treated within the same tertiary Pediatric Hematology–Oncology Department in Crete, Greece) were enrolled in the study. Self-reported HRQoL was assessed using the Short-Form Health Survey (SF-36). Sixty-five never-ill peers constituted the control group. Results: CCSs reach adolescence and young adulthood without significant deviations in HRQoL from their healthy peers. The presence and severity of late effects were significantly correlated with lower scores in physical health. The cancer type seems to play a pivotal role: Langerhans cell histiocytosis survivors displayed significantly lower scores in mental health, and brain tumor survivors scored substantially lower scores in physical functioning. Acute lymphoblastic leukemia survivors reported the highest scores in mental health. Age at diagnosis of neoplasia was negatively correlated with physical functioning. No significant sex differences were identified. Adherence to multiple healthy lifestyle behaviors (regular exercise, abstaining from alcohol consumption and smoking, and using sun protection) and active employment were correlated with significantly higher scores in mental health. Conclusions: Appropriate therapy and regular follow-up after treatment have led to improved clinical and social outcomes, as assessed by CCSs. More efforts are needed to increase awareness of avoiding harmful behaviors that raise the risk of late effects in this specific group.

## 1. Introduction

Following the advancements in childhood cancer treatment and survival rates, adolescents and young adults (AYAs) who are childhood cancer survivors (CCS) are a growing population who face distinctive challenges from pediatric and older survivors; hence, it is crucial to monitor the encountered issues with proper Patient Reported Outcome Measures (PROMs) [1]. Adolescence and young adulthood (referring to individuals aged between 15 and 39 years old) are critical periods in which several developmental, psychological, social, and physiological changes supervene with an expected endpoint: the transition toward independence. Inevitably, a cancer diagnosis interrupts normal development and personal growth, and in combination with the toxicities seen with cancer treatment, AYAs with cancer are negatively affected in multiple aspects of their physical, psychological, and social well-being [2,3]. The Childhood Cancer Survivor Study found that changes in childhood cancer therapy, such as reduced rates of cranial radiotherapy for acute lymphoblastic leukemia (ALL), abdominal radiotherapy for Wilms’ tumor (WT), chest radiotherapy for Hodgkin’s lymphoma (HL), and anthracycline exposure, resulted in significantly lower late mortality [4]. The same study reviewed health-related quality of life (HRQoL) and identified risk factors for psychological distress and poor HRQoL: female sex, lower educational level, unmarried status, annual household income (<USD 20,000), unemployment, lack of health insurance, presence of a significant medical condition, and treatment with cranial radiation and/or surgery [5]. A relevant report from the St. Jude Lifetime Cohort suggested that the association between healthy lifestyle factors (physical activity, healthy diet, maintaining an average weight, and abstaining from alcohol and tobacco) and HRQoL is cumulative, underlining the importance of adhering to multiple healthy lifestyle behaviors to improve poor HRQoL scores among long-term CCSs [6].

Long-term follow-up schedules are considered a prerequisite in CCSs to assess late effects and overall functioning in the years following childhood cancer. AYAs that are CCSs may report very good overall functioning in physical and general psychosocial domains (even equivalent to their peers who have not been ill), but plentiful generation-specific challenges exist. Psychological, social, and physical effects refer to AYAs’ complex physical and psychosocial development and to a range of lifestyle changes (such as higher education, employment, moving to their own home, romantic relationships, and parenting) together with transition challenges (anxiety about relapse, returning to school or work, and living with late effects or disability) [7]. PROMs facilitate patient participation in healthcare, enabling early issue identification, particularly in AYAs with developing autonomy. Evidence supports that PROMs enhance clinical management by improving communication, symptom monitoring, toxicity detection, and emotional well-being [1,8,9,10,11].

This study investigated the health status, HRQoL, and social outcomes in AYAs CCSs compared to healthy controls. This is the second study on AYAs CCSs of Greek descent in the literature (15 years ago), and the same HRQoL tools were used in the context of reproducibility [12]. The selection of the SF-36 was grounded in its extensive validation within CCSs, enabling meaningful comparisons with international literature. Although emerging PROMs such as EORTC QLQ-AYA, FACT-G, and PROMIS are gaining popularity in AYA survivorship research, the literature highlights significant heterogeneity and a deficiency in age-specific validation [1,13,14,15,16]. Despite the generic nature of the SF-36, it remains one of the most robust and widely used PROMs in this field, enabling meaningful cross-cohort comparisons and longitudinal assessments of survivorship outcomes. Our study, therefore, provides updated, region-specific data that complement recent European initiatives to harmonize outcome reporting in AYA survivors and address the relative scarcity of evidence from Mediterranean populations.

## 2. Materials and Methods

### 2.1. Study Design and Participants

The sample included 72 disease-free and off-treatment adolescents and young adults who had been diagnosed with childhood cancer in the Department of Pediatric Hematology–Oncology of the University Hospital of Heraklion, one of the six tertiary reference centers in Greece, providing services to children from Crete and the surrounding islands (with an estimated population of 650,000 inhabitants) for the last 35 years. They all met the following inclusion criteria: (a) diagnosis of cancer before the age of 18 years; (b) at least five years since diagnosis; (c) 2 years since completion of therapy; (d) age > 15 years at the time of the study; and (e) sufficient command of the Greek language. After obtaining the appropriate informed consent, the CCSs were given an anonymous questionnaire and asked to return it by mail within a month. Of the 72 invited CCSs, 62 consented to participation (86% response rate). The patient’s clinical records were reviewed to obtain some cancer-related variables, including the diagnosis, birth date, age at diagnosis, time since diagnosis, treatment variables, and late effects observed during the survey. Thus, CCS data combined both HRQoL self-reports and information from clinical records. The treatment of CCSs has been contingent on international protocols, while the intensity of treatment was defined as: (a) mild (e.g., only surgical resection or chemotherapy for a period of <6 months), (b) moderate (e.g., leukemia protocols low–medium risk), and (c) severe (e.g., aggressive treatment protocols of acute leukemia risk or hematopoietic stem cell transplantation) per previous reports [17]. Late effects have been defined according to the Common Terminology Criteria for Adverse Events v4.0 (CTCAEv4), which was initially developed by the National Cancer Institute [18]. For statistical analysis purposes, late effects were classified as mild (Grade 1), moderate (Grade 2), and severe (Grades 3 and 4).

After receiving the appropriate information and instructions, the questionnaires were also given to individuals from the participants’ broader environment who agreed to take part in the study’s control group. Eligible participants were individuals aged 15 to 35 years who reported no illnesses (acute or chronic) or disabilities. To prevent bias caused by close relationships with the CCSs, control group participants could be acquaintances but not family members or friends of the CCSs. A total of 65 subjects (57% response rate) completed anonymous questionnaires, all of whom were Greek nationals and formed the control group. For the control group, all data were self-reported without verification from medical records, and we acknowledge this as a limitation.

The study received approval from the Ethics Committee of the University Hospital of Heraklion and was carried out in line with the principles of the Declaration of Helsinki. Written informed consent was obtained from all participants. Parents or legal guardians of individuals aged 15 to 18 years were required to complete the appropriate form with their children.

### 2.2. SF-36

Self-reported HRQoL was assessed utilizing the SF-36 questionnaire (IQOLA SF-36 Greek standard version 1.0) [19]. The SF-36 is a generic self-report measure that offers a quick, comprehensive, and effective way of evaluating the quality of life of a person older than 14 years old. It contains 36 items that measure eight dimensions (subscales) of health status. In specific, the aspects that SF-36 assesses are the following: (i) physical functioning (PF) which assesses if health issues limit physical activities; (ii) role limitations caused by physical health problems (RP); (iii) role limitations caused by emotional health problems (RE); (iv) social functioning (SF) which assesses if physical or emotional problems interfere social activities; (v) mental health (MH) which assesses signs of depression, anxiety etc., (vi) energy–vitality (EV); (vii) bodily pain (BP); (viii) general health perception (GH) which explores the respondent’s estimation of their health. All subscales are scored on a 0 to 100 percentage scale, with a higher score indicating a higher level of health. In addition, from these eight subscales can be derived two summary scales: the Mental Component Summary Scale (MCS) and the Physical Component Summary Scale (PCS) [20]. The SF-36 Health Survey is one of the most frequently used tools (PROMs) and has been utilized by the most extensive studies on CCSs [1,5,21].

The SF-36 was accompanied by a specially designed questionnaire, which included questions on the participants’ education level (and that of their parents), marital and relationship status, living with family, occupational and self-rated financial status, health insurance, behavioral risk factors (alcohol consumption, tobacco use, substance abuse, and sun protection), and physical exercise. HRQoL scores were compared between the two groups and correlated with clinical and socio-demographic variables.

### 2.3. Data Collection and Analysis

All the data were gathered and categorized, and frequency counts were tabulated. Statistical analysis was performed using the IBM SPSS Statistics version 29.0.1.0 (IBM Corp., Armonk, NY, USA). The algorithm SF-36 Recode-Score PCS MCS.SPS (Greek standard version 1.0) was used for the assessment of the scales [19]. An *a priori* power analysis was conducted using G*Power 3.1.9.2 (Heinrich-Heine-Universität, Düsseldorf, Germany). A sample size of 62 CCSs and 65 healthy controls was sufficient to detect an odds ratio of 2.0 with 97.4% power at a two-tailed α = 0.05. This sample size also provides 80% power to detect a medium effect size (Cohen’s d = 0.50) in continuous outcome comparisons such as HRQoL scores. No missing value for any scale of the sample population was noted.

## 3. Results

### 3.1. Sample Characteristics

The study sample consisted of 62 CCSs and 65 individuals in the control group. Table 1 presents the sample socioeconomic, demographic, and health behavior traits by group and the respective two-sided Fisher’s Exact Test results. The only parameters that differed significantly between groups were the parental educational level (higher in the control group) and residing in a village (more common among CCSs). These variables were adjusted for each significant correlation found in this study. At the time of the survey, the mean age of CCSs was 21.9 (SD 4.7), while the mean age in the control group was 21.2 (SD 4.9) years old. The clinical characteristics of the CCS group are described in Table 2. The prevalence of neoplasia history follows the epidemiologic traits of childhood cancer worldwide, implying the representation of all significant childhood cancer types [22].

### 3.2. Internal Consistency and Reliability of Scales

Cronbach’s alpha internal consistency reliability coefficients ranged between 0.79 (social functioning; energy–vitality) and 0.82 (physical functioning, role limitations, physical component summary scale), satisfying in all cases the accepted criterion of 0.70 for comparisons between groups (Table 3). The correlations between scales ranged from 0.14 to 0.79 and were always less than their reliability coefficient, satisfying the primary criterion and confirming that each scale measures a distinct concept [20,23].

### 3.3. SF-36 Scores

Table 4 presents the mean scores of each subscale and summary scale for both groups. No significant differences were found between CCSs and controls (Mann-Whitney U Test). Effect sizes (Cohen’s d) indicated small differences across most subscales (|d| ≤ 0.21). Physical Functioning showed a small–to–moderate difference favoring controls (d = 0.38), although this did not reach statistical significance. Nevertheless, further data analysis revealed significant differences in SF-36 subscale scores among CCSs.

#### 3.3.1. Physical Functioning

- Survivors of central nervous system (CNS) tumors had the lowest scores (60; SD = 24.4) among CCSs, a statistically significant difference (*p* = 0.031) compared with ALL, the most prevalent type of childhood cancer (88; SD = 22.7).
- Scores of CCSs were negatively associated with the severity of late effects (*p* < 0.001), with significantly lower scores in survivors who experienced severe adverse events (50; SD = 23.2).

#### 3.3.2. Role Limitations Caused by Physical Health Problems

The scores of the severity of late effects differed significantly between groups (*p* = 0.008), with CCSs experiencing only mild adverse events scoring much higher (91.5; SD = 21.8) than those with non-mild adverse events.

#### 3.3.3. General Health Perception

The scores significantly differed between late effects classes (*p* = 0.049). CCSs who underwent mild adverse events reported significantly higher scores (74.6; SD = 21.4) than those who experienced moderate and severe adverse events (57.5; SD = 22.6; *p* = 0.017).

#### 3.3.4. Social Functioning

The cancer type was significantly associated with the scores in this subscale (*p* = 0.018). Survivors of Langerhans Cell Histiocytosis (LCH) were found with the lowest scores (50; SD = 25), and they differed significantly from survivors of ALL, non-Hodgkin lymphoma (NHL), and WT, who displayed higher mean scores (82.6 to 100; *p* = 0.007, *p* = 0.026, and *p* = 0.026, respectively).

#### 3.3.5. Mental Health

LCH survivors had the lowest scores (50.7; SD = 20.1), significantly lower than ALL survivors (72.1 with SD = 13.1; *p* = 0.015).

#### 3.3.6. Physical Component Summary

A significant score variation was recorded among the severity classes of late effects (*p* < 0.001). CCSs who encountered mild adverse events reported significantly higher scores (55.4; SD = 6.2) than those who experienced moderate and severe adverse events (45.4; SD = 11.2; *p* < 0.001).

#### 3.3.7. Mental Component Summary

LCH survivors displayed the lowest mean score (36.8; SD = 12), and they differed significantly from ALL and WT survivors, who displayed mean scores ranging between 48.1 and 57.7 (*p* = 0.047 and *p* = 0.044, respectively).

Age at the time of the study and sex have not been associated with any of the studied parameters. Neither a history of relapsed disease nor intense treatment was associated with lower scores in any of the subscales. Of note, age at diagnosis of neoplasia was negatively correlated with Physical Functioning among CCSs (r_S_ = −0.25; *p* = 0.05). Analysis of variance in the scores of all participants identified significant differences regarding: (i) occupational status and SF and MH (*p* = 0.011, and *p* = 0.024, respectively), (ii) maternal educational level and EV, RE, and MCS (*p* = 0.01, *p* = 0.028, and *p* = 0.011, respectively), (iii) paternal education and BP (*p* = 0.009), (iv) residence in a village and EV (*p* = 0.008), (v) physical exercise and EV (*p* = 0.043), (vi) smoking and GH (*p* = 0.026), and (vii) alcohol consumption and PF (*p* = 0.049). Further analysis of variance with tests of between-subjects effects replicated seven of the associations above, as demonstrated in Table 5.

Regarding CCSs, additional notable findings include higher RP and EV scores among participants who reported attending the gym (*p* = 0.032 and *p* = 0.033, respectively). Unemployment among controls was linked to lower physical functioning scores (*p* = 0.036). Overall, sun protection was significantly more common among female participants (*p* = 0.012), while alcohol consumption was more prevalent among males (*p* = 0.004). Notably, alcohol consumption rates were significantly lower among participants who still live with their parents (*p* = 0.021) and among singles (*p* = 0.003). Smoking was significantly more frequent among older participants (*p* = 0.027; Z = −2.213) and those who identified as being in a relationship (*p* < 0.001; OR 5.00 with 95% CI: 2.03–12.34).

## 4. Discussion

CCSs seem to reach adolescence and young adulthood without significant deviations in HRQoL from their never-ill peers (Table 4). Conversely, reports are suggesting that young adult CCSs may score better than the healthy standard population in physical health [24,25]. In terms of long-term survivorship, the time since the diagnosis (>10 years) does not seem to play a pivotal role in HRQoL among CCSs. However, lower scores for short-term survivorship are reasonably anticipated [26]. In our sample, a significant but weak association between low age at cancer diagnosis and higher Physical Functioning scores was calculated. Regarding sex differences, females are expected to report worse health than males (especially MCS), but this finding was not replicated by our results [5,6,19]. The education level of the participants was not associated with any of the subscales of SF-36, but higher maternal education has been linked with higher scores in EV, RE, and MCS for CCSs and relatively lower scores for the control group. Self-rated financial status and health insurance did not seem to have a meaningful effect on measured outcomes in this study. Contrarily, occupational status significantly affected SF and MH in this study, with active employment linked with higher scores. The CCSs had similar rates of committing to long-term relationships and living away from their families with their peers. Even though residing in a village was not equally distributed among the participants, living in small and large settlements seems to have a considerable effect on EV.

The prevalence of healthy lifestyle behaviors was comparable between CCSs and healthy controls. Decreased prevalence of alcohol consumption and tobacco use among Greek adolescents of the general population has been recorded in previous studies [27,28]. The association of smoking with GH was not replicated by significant between-subjects effects, while a weak association of alcohol use with PF was calculated. Regular physical exercise and use of sunscreen were not correlated with subscales’ scores, and they did not differ between CCSs and controls. Notably, cumulative healthy habits (regular exercise, no substance abuse, and use of sun protection) were correlated with MH, MCS, RE, and SF (*p* = 0.003, *p* < 0.001, *p* = 0.017, and *p* = 0.032, respectively). Similar results have been recently published for the adherence to multiple healthy lifestyle factors and HRQoL –based on the American Cancer Society’s Nutrition and Physical Activity Guidelines for Cancer Survivors [6].

A key finding of the current study is the significant impact of experiencing adverse events and late effects on the scores of the CCS group. The presence of these impediments was significantly correlated with lower PF, RP, GH, and PCS scores, and this effect was dependent on the severity of late effects. The importance of late effects in CCSs was reflected in low PF and GH in several studies in the field [29]. Their diversity and limited number prevent definitive links between specific late effects and any of the studied scores. Another pertinent finding was that the cancer type itself was associated with scores in SF-36. Survivors of brain tumors had the lowest PF. This result is in agreement with a previous report on CCSs, which concluded that having had CNS or bone cancer is independently associated with poorer HRQoL in the physical dimensions [30]. There is only one more study in the literature to report SF-36 scores in LCH survivors, who are traditionally treated within Pediatric Hematology–Oncology departments. LCH survivors displayed significantly lower scores in SF, MH, and MCS. Prior studies reported lower overall health scores in SF-36 among LCH survivors, whose chronic and relapsing disease course, with frequent skeletal morbidity, may partly explain this result [31,32].

Unlike the Scandinavian and Central European countries, studies on AYAs CCSs from the Mediterranean and Balkan regions are lacking. Only two studies from these geographic regions reported SF-36 results, but they concern the parents of AYAs CCSs [33,34]. A study from Italy reported good overall HRQoL using the Health Utilities Index Mark III (HUI3) questionnaire in AYAs CCSs. In the latter study, a greater probability of impaired HRQoL was identified for females, survivors of CNS tumors, retinoblastoma, and bone tumors, and persons diagnosed before ten years of age [35].

This is the second study in the literature that refers to Greek CCSs. The first study on Greek CCSs was conducted in Greece’s capital and published in 2009. The results of that study were parity with the current study, suggesting that the level of care for children with cancer in Greece is comparable between the capital and remote regions [12]. Two differences with the previous study were identified: (i) No differences in Bodily Pain were detected in our sample, and (ii) No positive correlation between age at diagnosis and RP, RE, and EV scores in CCSs was noted. Contrary to the latter findings, which imply that long-term difficulties in functional ability may be more prominent in CCSs diagnosed during infancy or early childhood, the current study (along with others) supports the theory that low age at cancer diagnosis might link with restored scores in Physical Functioning after > 10 years—even though the risk for late effects increases [5,36]. An explanation for this inconsistency could be the improvement in preventive measures and the provision for lower treatment-related toxicities during the last decade.

Transplanted patients are expected to display worse Physical Component Summary scores, while psychological factors such as resilience and depression play key roles in these patients [37,38]. Transplanted patients in our study showed no significant differences compared to other CCSs; however, their small sample size (n = 8) does not warrant safe conclusions. Nevertheless, the experience of worse late effects is a well-established risk factor for low HRQoL according to seven relevant SF-36 studies [39].

This study’s strengths include a high response rate among the CCS group and a representative sample of CCSs. In addition, completed questionnaires were mailed back to the Department, making the responses more homogeneous and objective, and refraining from entering the hospital setting, which may have provoked some reactions due to unpleasant memories. This study has several limitations. Control group data were exclusively self-reported, while CCSs benefited from validation against clinical records. Subgroup sample sizes, particularly for LCH survivors and transplanted patients, were small, limiting the strength of conclusions. The cross-sectional design precludes causal inference, and some participants were relatively early survivors; longitudinal follow-up is required to capture changes over time. Moreover, the CCS group consisted of 45% ALL survivors, which implies an expectation of a reasonably good HRQoL post-treatment [40]. Differences in the CCS groups (diagnosis or follow-up interval) and the control group for comparisons (siblings, healthy peers, or the general population) can result in conflicting results, precluding reproducibility [41].

This study’s findings have significant implications for clinical practice and policymaking. Healthcare professionals should assess and longitudinally monitor the HRQoL of AYAs, especially when transitioning from pediatric to adult healthcare systems. Policymakers and support organizations should prioritize awareness campaigns to increase knowledge of support resources (resuming studies or work, financial aid, and social welfare programs) and improve access to healthcare for AYAs (including fertility issues and genetic counseling).

## 5. Conclusions

HRQoL in CCSs may differ only slightly from that of their siblings and the general population, but these differences can become more pronounced when considering specific cancer types and late effects, especially when different control groups are used. Additionally, HRQoL may depend on the time since diagnosis. The current study found significantly lower HRQoL scores among CCSs with late effects other than mild, as well as among LCH and brain tumor survivors. In contrast, we found higher scores in participants who follow healthy lifestyle behaviors. Occupational status and resilience factors also appear to play key roles in HRQoL. For clinicians, the take-home message is the importance of risk-based, long-term follow-up tailored to treatment exposures, proactive screening for psychosocial difficulties, and the encouragement of healthy lifestyle behaviors that can mitigate late effects. Future studies should follow survivors longitudinally, include multicenter Mediterranean and Balkan cohorts, and integrate AYA-specific PROMs to capture the evolving needs of this population. Results from the PanCareLIFE study, which recruited over 10,000 CCSs, are highly anticipated to shed light on differences among European countries using the SF-36 tool [42].

## Figures and Tables

**Table 1:** Sociodemographic characteristics and health behaviors of the participants.

	CCSs n (%)	Control Group n (%)	*p*
Age group (years)			0.374
15–17	10 (16.1)	16 (24.6)	
18–24	35 (56.5)	29 (44.6)	
25–35	17 (27.4)	20 (30.8)	
Sex			0.155
Female	31 (50)	41 (63.1)	
Male	31 (50)	24 (36.9)	
Education			0.876
University or Higher Technological Institution	28 (45.2)	27 (41.5)	
Vocational School or High School	28 (45.1)	30 (46.2)	
Secondary School or Primary School	6 (9.7)	8 (12.3)	
Paternal education			<0.001
University or Higher Technological Institution	10 (16.1)	22 (33.8)	
Vocational School or High School	18 (29)	31 (47.7)	
Secondary School or Primary School	34 (54.8)	12 (18.5)	
Maternal education			0.007
University or Higher Technological Institution	12 (19.4)	22 (33.8)	
Vocational School or High School	23 (37.1)	31 (47.7)	
Secondary School or Primary School	27 (43.5)	12 (18.5)	
Current job and/or studies			0.248
Regular/casual work	19 (30.6)	11 (16.9)	
Studies	27 (43.5)	38 (58.5)	
Studies and work	4 (6.5)	5 (7.7)	
No work or studies	12 (19.4)	11 (16.9)	
Place of living			0.011
Village	15 (24.2)	4 (6.2)	
Small town	7 (11.3)	6 (9.2)	
City	40 (64.5)	55 (84.6)	
Relationship status			0.843
Married or partnered	18 (29)	17 (26.2)	
Single	44 (71)	48 (73.8)	
Living with family			0.129
Yes	45 (72.6)	55 (84.6)	
No	17 (27.4)	10 (15.4)	
Self-rated financial status			0.665
Poor	3 (4.8)	1 (1.5)	
Ordinary	42 (67.7)	45 (69.2)	
Good	17 (27.4)	19 (29.2)	
Health insurance			0.675
Public or private	59 (95.2)	63 (96.9)	
Uninsured	3 (4.8)	2 (3.1)	
Physical exercise			0.245
Yes (≥once a week)	40 (64.5)	49 (75.4)	
Never or scarcely	22 (35.5)	16 (24.6)	
Smoking			1.000
Yes	13 (21)	14 (21.5)	
No	49 (79)	51 (78.5)	
Alcohol consumption			0.642
Yes	12 (19.4)	10 (15.4)	
No	50 (80.6)	55 (84.6)	
Sun protection			0.497
Never	5 (8.1)	6 (9.2)	
Sometimes	35 (56.5)	30 (46.2)	
Always	22 (35.5)	29 (44.6)	

**Table 2:** Clinical characteristics of the CCS group.

Mean Age at Diagnosis ± SD (Years)	8.7 ± 5.1
Median age at the time of study (years)	21 (range: 15–30.9)
Median time since diagnosis (years)	14.7 (range: 3.4–26.6)
	N (%)
Neoplasia	
Acute Lymphoblastic Leukemia	28 (45.2)
Acute Myeloid Leukemia	4 (6.5)
Non-Hodgkin Lymphoma	3 (4.8)
Hodgkin Lymphoma	8 (12.9)
CNS tumor	4 (6.5)
Wilms’ Tumor	3 (4.8)
Neuroblastoma	4 (6.5)
Germ Cell Tumors	4 (6.5)
Osteosarcoma	1 (1.6)
Langerhans Cell Histiocytosis	3 (4.8)
Intensity of treatment	
Mild	16 (25.8)
Moderate	20 (32.3)
Severe	26 (41.9)
Adverse events	
Grade 1	50 (80.6)
Grade 2	7 (11.3)
Grade 3 or 4	5 (8.1)
Relapse	
No	59 (95.2)
Yes	3 (4.8)
Transplantation	
No	54 (87.1)
Yes	8 (12.9)

**Table 3:** Reliability coefficients and inter-scale correlations.

	PF	RP	BP	GH	EV	SF	RE	MH	PCS	MCS
**PF**	0.82									
**RP**	0.37	0.82								
**BP**	0.28	0.41	0.80							
**GH**	0.39	0.28	0.42	0.81						
**EV**	0.46	0.25	0.52	0.49	0.79					
**SF**	0.39	0.31	0.46	0.42	0.56	0.79				
**RE**	0.29	0.45	0.31	0.34	0.37	0.50	0.81			
**MH**	0.24	0.14	0.39	0.30	0.65	0.58	0.44	0.80		
**PCS**	0.51	0.63	0.51	0.55	0.25	0.10	0.07	−0.14	0.82	
**MCS**	0.20	0.17	0.30	0.27	0.61	0.68	0.76	0.79	−0.21	0.80

Cronbach’s a coefficients are presented in the diagonal.

**Table 4:** Comparison of the subscales’ scores and the summary scales between the CCS and the control group.

	CCSs Mean (SD)	Control Group Mean (SD)	*p*
Physical Functioning (PF)	87.8 (19.4)	93.5 (9.4)	0.113
Role Limitations caused by Physical Health Problems (RP)	87.5 (26.3)	82.7 (27.9)	0.22
Bodily Pain (BP)	84.2 (22.6)	80 (23.6)	0.249
General Health Perception (GH)	71.3 (22.5)	75.2 (14.2)	0.757
Energy–Vitality (EV)	73.2 (17.6)	70.9 (20.8)	0.437
Social Functioning (SF)	81 (20.2)	80.2 (21.5)	0.899
Role Limitations caused by Emotional Health Problems (RE)	74.2 (33.3)	73.3 (34)	0.966
Mental Health (MH)	70.6 (18.9)	71.8 (19.9)	0.931
Physical Component Summary (PCS)	53.4 (8.3)	53.8 (5.8)	0.597
Mental Component Summary (MCS)	48.1 (10.3)	47.7 (11.2)	0.963

**Table 5:** Significant between-subjects effects in subscale scores.

	CCS Mean (SD)	Control Group Mean (SD)	*p*
Social Functioning and Occupational Status			
Work	86.8 (17.4)	87.5 (14.8)	0.011
Studies	75.5 (22.6)	84.9 (15.7)	
Work and studies	84.4 (12)	70 (25.9)	
No work or studies	83.3 (19.5)	61.4 (31.4)	
Mental Health and Occupational Status			
Work	73.7 (18.1)	78.2 (15.7)	0.024
Studies	69.3 (20.3)	77.4 (12.6)	
Work and studies	75 (18.9)	66.4 (17.8)	
No work or studies	67 (18.1)	48.7 (28.3)	
Energy–Vitality and Maternal Education			
University or Technological Institution	77.1 (16)	61.1 (18.9)	0.01
High/Vocational School	71.7 (18.9)	72.3 (20)	
Illiterate/Primary/Secondary School	72.8 (17.6)	85.4 (17.6)	
Role Limitations by Emotional Health Problems and Maternal Education			
University or Technological Institution	86.1 (26.4)	63.6 (35.5)	0.028
High/Vocational School	76.8 (32.5)	74.2 (34.1)	
Illiterate/Primary/Secondary School	66.7 (35.8)	88.9 (25.9)	
Mental Component Summary and Maternal Education			
University or Technological Institution	50.7 (6.7)	42.9 (11.6)	0.011
High/Vocational School	48.3 (11.6)	48.2 (10.7)	
Illiterate/Primary/Secondary School	46.8 (10.6)	55.1 (7.3)	
Energy–Vitality and Residence			
City	74.4 (16.7)	69 (19.6)	0.008
Small Town	78.6 (17.5)	70.8 (27.8)	
Village	67.7 (19.8)	97.5 (5)	
Physical Functioning and Alcohol			
Yes	92.9 (10.1)	87 (17.7)	0.049
No	86.6 (20.9)	94.7 (6.6)	

## Data Availability

The data presented in this study are available from the corresponding author upon request.

## Abbreviations

The following abbreviations are used in this manuscript:

AYA	Adolescents and Young Adults
BP	Bodily pain
CCS	Childhood Cancer Survivors
CNS	Central Nervous System
CTCAE	Common Terminology Criteria for Adverse Events
EV	Energy–Vitality
GH	General health perception
HL	Hodgkin’s Lymphoma
HRQoL	Health-Related Quality of Life
LCH	Langerhans Cell Histiocytosis
MCS	Mental Component Summary Scale
MH	Mental health
NHL	Non-Hodgkin Lymphoma
PCS	Physical Component Summary Scale
PF	Physical functioning
PROM	Patient Reported Outcome Measures
RE	Role limitations caused by emotional health problems
RP	Role limitations caused by physical health problems
SD	Standard deviation
SF	Social functioning
SF-36	36-Item Short-Form Health Survey
WT	Wilms’ Tumor

## References

[1] Engstrom T., Tanner S., Lee W.R., Forbes C., Walker R., Bradford N., Pole J.D. (2023). Patient Reported Outcome Measure Domains and Tools Used among Adolescents and Young Adults with Cancer: A Scoping Review. Crit. Rev. Oncol. Hematol..

[2] Barnett M., McDonnell G., DeRosa A., Schuler T., Philip E., Peterson L., Touza K., Jhanwar S., Atkinson T.M., Ford J.S. (2016). Psychosocial Outcomes and Interventions among Cancer Survivors Diagnosed during Adolescence and Young Adulthood (AYA): A Systematic Review. J. Cancer Surviv..

[3] Bradford N.K., McDonald F.E.J., Bibby H., Kok C., Patterson P. (2022). Psychological, Functional and Social Outcomes in Adolescent and Young Adult Cancer Survivors over Time: A Systematic Review of Longitudinal Studies. Psychooncology.

[4] Armstrong G.T., Chen Y., Yasui Y., Leisenring W., Gibson T.M., Mertens A.C., Stovall M., Oeffinger K.C., Bhatia S., Krull K.R. (2016). Reduction in Late Mortality among 5-Year Survivors of Childhood Cancer. N. Engl. J. Med..

[5] Zeltzer L.K., Recklitis C., Buchbinder D., Zebrack B., Casillas J., Tsao J.C.I., Lu Q., Krull K. (2009). Psychological Status in Childhood Cancer Survivors: A Report from the Childhood Cancer Survivor Study. J. Clin. Oncol..

[6] Zhang F.F., Hudson M.M., Huang I.-C., Bhakta N., Ness K.K., Brinkman T.M., Klosky J., Lu L., Chen F., Ojha R.P. (2018). Lifestyle Factors and Health-Related Quality of Life in Adult Survivors of Childhood Cancer: A Report from the St. Jude Lifetime Cohort. Cancer.

[7] Shinohara Y., Morino T., Shimoura K., Niu Q., Mukaiyama K., Chen C., Matsumura N., Shimizu H., Tabata A., Hanai A. (2023). Comparison of Psychological Quality of Life Between Long-Term Survivors of Childhood Cancer and Their Families. J. Adolesc. Young Adult Oncol..

[8] Janssen S.H.M., Vlooswijk C., Bijlsma R.M., Kaal S.E.J., Kerst J.M., Tromp J.M., Bos M.E.M.M., van der Hulle T., Lalisang R.I., Nuver J. (2025). Health-Related Quality of Life of Long-Term Adolescent and Young Adult (AYA) Cancer Survivors Compared to a Matched Normative Population: Results of the SURVAYA Study. J. Cancer Surviv..

[9] de Ligt K.M., Hommes S., Vromans R.D., Boomstra E., van de Poll L.V., Krahmer E.J. (2025). Improving the Implementation of Patient-Reported Outcome Measure in Clinical Practice: Tackling Current Challenges With Innovative Digital Communication Technologies. J. Med. Internet Res..

[10] Lowry V., Tremblay-Vaillancourt V., Beaupré P., Poirier M.-D., Perron M.-È., Bernier J., Morin A., Cormier C., Haggerty J., Ahmed S. (2024). How Patient-Reported Outcomes and Experience Measures (PROMs and PREMs) Are Implemented in Healthcare Professional and Patient Organizations? An Environmental Scan. J. Patient-Rep. Outcomes.

[11] Churruca K., Pomare C., Ellis L.A., Long J.C., Henderson S.B., Murphy L.E.D., Leahy C.J., Braithwaite J. (2021). Patient-reported Outcome Measures (PROMs): A Review of Generic and Condition-specific Measures and a Discussion of Trends and Issues. Health Expect..

[12] Servitzoglou M., Papadatou D., Tsiantis I., Vasilatou-Kosmidis H. (2009). Quality of Life of Adolescent and Young Adult Survivors of Childhood Cancer. J. Pediatr. Nurs..

[13] Siembida E.J., Reeve B.B., Zebrack B.J., Snyder M.A., Salsman J.M. (2021). Measuring Health-Related Quality of Life in Adolescent and Young Adult Cancer Survivors with the National Institutes of Health Patient-Reported Outcomes Measurement Information System^®^: Comparing Adolescent, Emerging Adult, and Young Adult Survivor Perspectives. Psychooncology.

[14] Murphy K.M., Siembida E., Lau N., Berkman A., Roth M., Salsman J.M. (2023). A Systematic Review of Health-Related Quality of Life Outcomes in Psychosocial Intervention Trials for Adolescent and Young Adult Cancer Survivors. Crit. Rev. Oncol. Hematol..

[15] Tanner S., Engstrom T., Forbes C., Patel D., Lee W.R., Walker R., Bradford N., Pole J.D. (2024). Physical Function Patient-Reported Outcomes among Adolescent and Young Adult Cancer Survivors: A Systematic Review. Cancer Med..

[16] Bradford N., Pitt E., Rumble S., Cashion C., Lockwood L., Alexander K. (2022). Persistent Symptoms, Quality of Life, and Correlates with Health Self-Efficacy in Adolescent and Young Adult Survivors of Childhood Cancer. J. Adolesc. Young Adult Oncol..

[17] Meeske K.A., Ruccione K., Globe D.R., Stuber M.L. (2001). Posttraumatic Stress, Quality of Life, and Psychological Distress in Young Adult Survivors of Childhood Cancer. Oncol. Nurs. Forum.

[18] Miller T.P., Fisher B.T., Getz K.D., Sack L., Razzaghi H., Seif A.E., Bagatell R., Adamson P.C., Aplenc R. (2019). Unintended Consequences of Evolution of the Common Terminology Criteria for Adverse Events. Pediatr. Blood Cancer.

[19] Pappa E., Kontodimopoulos N., Niakas D. (2005). Validating and Norming of the Greek SF-36 Health Survey. Qual. Life Res..

[20] Ware J.E., Gandek B. (1998). Overview of the SF-36 Health Survey and the International Quality of Life Assessment (IQOLA) Project. J. Clin. Epidemiol..

[21] Howell C.R., Bjornard K.L., Ness K.K., Alberts N., Armstrong G.T., Bhakta N., Brinkman T., Caron E., Chemaitilly W., Green D.M. (2020). Cohort Profile: The St. Jude Lifetime Cohort Study (SJLIFE) for Paediatric Cancer Survivors. Int. J. Epidemiol..

[22] Wu Y., Deng Y., Wei B., Xiang D., Hu J., Zhao P., Lin S., Zheng Y., Yao J., Zhai Z. (2022). Global, Regional, and National Childhood Cancer Burden, 1990–2019: An Analysis Based on the Global Burden of Disease Study 2019. J. Adv. Res..

[23] Reulen R.C., Zeegers M.P., Jenkinson C., Lancashire E.R., Winter D.L., Jenney M.E., Hawkins M.M. (2006). The Use of the SF-36 Questionnaire in Adult Survivors of Childhood Cancer: Evaluation of Data Quality, Score Reliability, and Scaling Assumptions. Health Qual Life Outcomes.

[24] Pemberger S., Jagsch R., Frey E., Felder-Puig R., Gadner H., Kryspin-Exner I., Topf R. (2005). Quality of Life in Long-Term Childhood Cancer Survivors and the Relation of Late Effects and Subjective Well-Being. Support. Care Cancer.

[25] Rourke M.T., Kazak A.E., Schwartz C.L., Hobbie W.L., Constine L.S., Ruccione K.S. (2005). Psychological Aspects of Long-Term Survivorship. Survivors of Childhood and Adolescent Cancer: A Multidisciplinary Approach.

[26] Rueegg C.S., Michel G., Wengenroth L., von der Weid N.X., Bergstraesser E., Kuehni C.E., Swiss Paediatric Oncology Group (SPOG) (2012). Physical Performance Limitations in Adolescent and Adult Survivors of Childhood Cancer and Their Siblings. PLoS ONE.

[27] Arvanitidou M., Tirodimos I., Kyriakidis I., Tsinaslanidou Z., Seretopoulos D., Dardavessis T. (2008). Cigarette Smoking among Adolescents in Thessaloniki, Greece. Int. J. Public Health.

[28] Arvanitidou M., Tirodimos I., Kyriakidis I., Tsinaslanidou Z., Seretopoulos D. (2007). Decreasing Prevalence of Alcohol Consumption Among Greek Adolescents. Am. J. Drug Alcohol Abus..

[29] Ishida Y., Honda M., Kamibeppu K., Ozono S., Okamura J., Asami K., Maeda N., Sakamoto N., Inada H., Iwai T. (2011). Social Outcomes and Quality of Life of Childhood Cancer Survivors in Japan: A Cross-Sectional Study on Marriage, Education, Employment and Health-Related QOL (SF-36). Int. J. Hematol..

[30] Maunsell E., Pogany L., Barrera M., Shaw A.K., Speechley K.N. (2006). Quality of Life among Long-Term Adolescent and Adult Survivors of Childhood Cancer. J. Clin. Oncol..

[31] Lau L.M.S., Stuurman K., Weitzman S. (2008). Skeletal Langerhans Cell Histiocytosis in Children: Permanent Consequences and Health-Related Quality of Life in Long-Term Survivors. Pediatr. Blood Cancer.

[32] Sveijer M., Gavhed D., Hertzberg H., Zander E., Henter J.-I., von Bahr Greenwood T. (2025). Quality of Life, Fatigue, Depression, Attention Deficits, and Pain after Childhood Langerhans Cell Histiocytosis. Blood Neoplasia.

[33] Tapak L., Cheraghi F., Sadeghi A., Shirmohammadi N., Feizybarnaji A. (2022). Usefulness of the SF-36 Health Survey Questionnaire in Screening for Health-Related Quality of Life among Parents of Children with Cancer: Latent Profile Analysis. J. Prev. Med. Hyg..

[34] Sezgin G., Yaylaci A., Unal I. (2023). Quality of Life in Parents of Turkish and Syrian Pediatric Bone Marrow Transplant and Oncology Patients. Niger. J. Clin. Pract..

[35] Alessi D., Dama E., Barr R., Mosso M.L., Maule M., Magnani C., Pastore G., Merletti F. (2007). Health-Related Quality of Life of Long-Term Childhood Cancer Survivors: A Population-Based Study from the Childhood Cancer Registry of Piedmont, Italy. Eur. J. Cancer.

[36] Michel G., Greenfield D.M., Absolom K., Ross R.J., Davies H., Eiser C. (2009). Follow-up Care after Childhood Cancer: Survivors’ Expectations and Preferences for Care. Eur. J. Cancer.

[37] Bernard F., Auquier P., Herrmann I., Contet A., Poiree M., Demeocq F., Plantaz D., Galambrun C., Barlogis V., Berbis J. (2014). Health Status of Childhood Leukemia Survivors Who Received Hematopoietic Cell Transplantation after BU or TBI: An LEA Study. Bone Marrow Transplant..

[38] Tremolada M., Bonichini S., Taverna L., Basso G., Pillon M. (2018). Health-Related Quality of Life in AYA Cancer Survivors Who Underwent HSCT Compared with Healthy Peers. Eur. J. Cancer Care.

[39] Vetsch J., Wakefield C.E., Robertson E.G., Trahair T.N., Mateos M.K., Grootenhuis M., Marshall G.M., Cohn R.J., Fardell J.E. (2018). Health-Related Quality of Life of Survivors of Childhood Acute Lymphoblastic Leukemia: A Systematic Review. Qual. Life Res..

[40] Godoy-Casasbuenas N., de Vries E. (2022). Self-Reported Health Problems and Quality of Life in a Sample of Colombian Childhood Cancer Survivors: A Descriptive Cross-Sectional Study. Cancers.

[41] van Erp L.M.E., Maurice-Stam H., Kremer L.C.M., Tissing W.J.E., van der Pal H.J.H., de Vries A.C.H., van den Heuvel-Eibrink M.M., Versluys B.A.B., Loonen J.J., Bresters D. (2021). Health-Related Quality of Life in Dutch Adult Survivors of Childhood Cancer: A Nation-Wide Cohort Study. Eur. J. Cancer.

[42] Byrne J., Grabow D., Campbell H., O’Brien K., Bielack S., Am Zehnhoff-Dinnesen A., Calaminus G., Kremer L., Langer T., van den Heuvel-Eibrink M.M. (2018). PanCareLIFE: The Scientific Basis for a European Project to Improve Long-Term Care Regarding Fertility, Ototoxicity and Health-Related Quality of Life after Cancer Occurring among Children and Adolescents. Eur. J. Cancer.

